# A Novel Mobile Phone App Intervention With Phone Coaching to Reduce Symptoms of Depression in Survivors of Women’s Cancer: Pre-Post Pilot Study

**DOI:** 10.2196/15750

**Published:** 2020-02-06

**Authors:** Philip I Chow, Fabrizio Drago, Erin M Kennedy, Wendy F Cohn

**Affiliations:** 1 University of Virginia Charlottesville, VA United States

**Keywords:** mobile apps, mental health, mHealth, women, cancer survivors

## Abstract

**Background:**

Psychological distress is a major issue among survivors of women’s cancer who face numerous barriers to accessing in-person mental health treatments. Mobile phone app–based interventions are scalable and have the potential to increase access to mental health care among survivors of women’s cancer worldwide.

**Objective:**

This study aimed to evaluate the acceptability and preliminary efficacy of a novel app-based intervention with phone coaching in a sample of survivors of women’s cancer.

**Methods:**

In a single-group, pre-post, 6-week pilot study in the United States, 28 survivors of women’s cancer used iCanThrive, a novel app intervention that teaches skills for coping with stress and enhancing well-being, with added phone coaching. The primary outcome was self-reported symptoms of depression (Center for Epidemiologic Studies Depression Scale). Emotional self-efficacy and sleep disruption were also assessed at baseline, 6-week postintervention, and 4 weeks after the intervention period. Feedback obtained at the end of the study focused on user experience of the intervention.

**Results:**

There were significant decreases in symptoms of depression and sleep disruption from baseline to postintervention. Sleep disruption remained significantly lower at 4-week postintervention compared with baseline. The iCanThrive app was launched a median of 20.5 times over the intervention period. The median length of use was 2.1 min. Of the individuals who initiated the intervention, 87% (20/23) completed the 6-week intervention.

**Conclusions:**

This pilot study provides support for the acceptability and preliminary efficacy of the iCanThrive intervention. Future work should validate the intervention in a larger randomized controlled study. It is important to develop scalable interventions that meet the psychosocial needs of different cancer populations. The modular structure of the iCanThrive app and phone coaching could impact a large population of survivors of women’s cancer.

## Introduction

### Background

A large body of literature demonstrates that survivors of cancers that almost exclusively affect women (ie, those who have completed treatment for breast, endometrial, and gynecologic cancers) have large and unmet psychosocial care needs [1-5]. Studies have found that during primary cancer treatment, the emotional needs of patients are often neglected [6-8], and this pattern continues into survivorship. Depression, which affects between 10% and 25% of survivors of women’s cancer [4,9,10], is perhaps the most studied psychosocial effect of cancer treatment. For example, among individuals with breast cancer, those who report a clinical level of depression report 2½ times as many unmet psychosocial needs compared with those without significant depression [2]. Untreated symptoms of depression can lead to poor quality of life [11], increased mortality [12,13], and high economic costs [14]. Furthermore, studies find that depression is closely linked with other health behaviors, such as sleep disturbance and difficulty in regulating emotions [15,16]. It is, therefore, imperative to develop and test effective, scalable, and accessible psychosocial interventions to meet the growing needs of survivors of women’s cancer.

Interventions that emphasize skills acquisition, such as cognitive behavioral therapy and acceptance-based therapies (eg, mindfulness), have been shown to effectively reduce symptoms of depression in cancer survivors [17-20]. However, numerous barriers prevent them from receiving adequate in-person treatment [21]: high financial cost [22], high time investment [23], social stigma [24], and a severe shortage of trained psychotherapists [25-27]. Combined, these barriers lead to almost half of the survivors to report unmet psychosocial care needs [3,8,28-31]. For example, although psychosocial interventions have been found to reduce depressive symptoms for early stage breast cancer patients [32,33], more recent research has identified the need to address depression symptoms up to 5 years following primary cancer treatment [34,35].

Mobile phone apps are frequently cited as a way of extending cost-effective care [36,37]. The percentage of US adults who own an internet-enabled mobile phone has steadily increased from 35% in 2011 to 77% in 2018, including 73% of US adults aged 50 to 64 years [38]. Numerous studies have demonstrated the efficacy of app-based interventions in reducing mood-related symptoms in the general population [37,39-42]. App-based interventions decrease barriers associated with traditional in-person interventions for cancer survivors because treatment is affordable, can be made more readily available by removing logistical issues (ie, travel and scheduling), can offer an efficient use of time (ie, no delays to begin treatment and self-pacing), and is no longer limited by proximity to available psychotherapists. However, despite high demand from survivors of women’s cancer for receiving care through digital means [43-46], few interventions specifically target mental health and well-being in this rapidly growing population. Empirical reviews of apps in cancer [47,48] fail to identify any publicly available mental health interventions for survivors of women’s cancer.

### Coaching to Enhance Engagement to Digital Interventions

Despite the promise of mobile interventions to increase scalability and access to mental health care, engagement is a major problem [49]. Engagement is necessary for treatment success, as a dose-response relationship has been observed in psychological treatment broadly [50] and in digital health interventions [51,52]. Numerous studies have found that poor engagement is a widespread concern across digital interventions, leading to dropout rates often as high as 50% [49,53-55], and meta-analyses have found that dropout rates are particularly high among depressed participants [56-58]. Similar rates of dropout have been noted in Web- and app-based interventions for cancer populations as in the general population [59-61]. Thus, despite the need for app-based interventions to reduce the impact of symptoms of depression in survivors of women’s cancer, existing interventions have not been designed to optimize engagement, which can restrict outcomes.

A growing amount of work has evaluated human support strategies to promote engagement, such as phone coaching [39,62,63], that may be particularly useful for promoting engagement among women. Studies have found that compared with men, women use health services more [64], prefer mediated social interaction [65], and tend to favor dyadic social relationships [66]. These findings indicate that integrating human support with an app-based intervention could be an effective strategy to increase engagement among survivors of women’s cancer. The Efficiency Model of Support [63] is a model for how to provide a provision of support to users of a digital health app. Specifically, the model highlights 5 ways that users might fail to benefit from a health app. These include issues related to the usability of the program, fit of the app to meet one’s needs, knowledge of how to use the program, and implementation failures [63]. On the basis of model, the role of the coach is to support users in using and benefiting from an app-delivered intervention, by identifying and targeting factors (eg, lack of understanding of how to use the app or how the app can improve daily life) that may lead users to fail to benefit from the program, and providing support to overcome those factors. This study paired an app intervention with phone coaching to enhance engagement and thus promote outcomes.

### This Study

In a sample of survivors of women’s cancer in the United States who completed their active cancer treatment within the last 5 years, the primary goals of this study were to evaluate the acceptability and preliminary efficacy of a novel app-based intervention (iCanThrive) over a 6-week period and to inform the sample size for a larger trial [67]. The iCanThrive app was designed as a clinical intervention that teaches skills for coping, reducing distress, and promoting strengths (see description below in section titled iCanThrive App). We define acceptability similar to others [68] as a multifaceted construct that pertains to how much users of an intervention find it to be appropriate and the degree to which it meets their needs [68]. In this study, acceptability was evaluated through the user experience domains of usefulness, ease of use, ease of learning, and satisfaction of using the app. Symptoms of depression were assessed at baseline, 6 weeks after the intervention (postintervention), and 4 weeks after the intervention period (4-week follow-up). Owing to the positive relationship between depression and sleep disturbance [16] and because iCanThrive teaches skills for regulating affect, additional outcomes assessed were self-reported sleep disturbance and emotional self-efficacy. Acceptability data were collected at the postintervention assessment.

## Methods

### Overview

This was a single-group, 6-week, pre-post pilot study design among survivors of women’s cancer in the United States who completed their active cancer treatment in the last 5 years. The decision to use a 6-week duration was based on the duration of brief face-to-face psychotherapy (typically 6-8 weeks) as well as prior reviews of mobile health (mHealth) studies finding that the duration of app-based interventions ranges between 6 days and 8 weeks [69]. Acceptability was assessed at the postintervention assessment. Self-reported symptoms of depression, emotional self-efficacy, and sleep disturbance were administered at baseline, postintervention, and 4-week follow-up.

### Participants and Procedure

A total of 28 survivors of women’s cancer (mean age 59.6 years, SD 10.5) were recruited from a community research cohort in the United States that included patients from 2 large regional cancer centers in the United States with catchment areas serving rural and nonrural communities. Women aged older than 18 years were eligible to join the cohort if they had received a diagnosis of stage 1, 2, or 3 breast, cervical, ovarian, or endometrial/uterine cancer and if they were more than 6 months from completing their active cancer treatment (surgery, chemotherapy, or radiation). Women in this study reported being diagnosed with the following types of cancer (they could choose more than 1): breast (n=13), bladder (n=1), cervical (n=1), ovarian (n=7), and endometrial (n=9). On average, the duration between their last cancer treatment and the beginning of study enrollment was 2.5 years (range 1.4-3.9 years). Most participants self-identified as white (26/28, 94%), followed by black (1/28, 3%) and multiracial (1/28, 3%). Rural-urban commuting area (RUCA) codes version 2.0 from the US Department of Agriculture were used to evaluate the geographic characteristics of the sample. RUCA codes range from 1 (most metropolitan) to 10 (most rural). Participants hailed from a range of locations, with 64% (18/28) living in an area characterized as metropolitan (RUCA 1-3), 18% (5/28) living in an area characterized as micropolitan (RUCA 4-6), and 18% (5/28) living in an area characterized as rural (RUCA 7-10).

A target sample size of 30 was chosen based on sample size recommendations from prior work for a pilot study [67]. This sample size is sufficient for detecting a large effect size for change in depression symptoms, at 80% power, although 1 purpose of conducting this pilot study was to collect initial data to inform a sample size calculation for a larger trial [67]. To reduce barriers for participation, inclusion criteria were limited to the following: (1) woman cancer survivor who completed their active cancer treatment in the last 5 years, (2) aged at least 18 years, and (3) owned a mobile phone or is willing to carry one around if provided. As this pilot study evaluated a brand-new app intervention, given the resource demands of prescreening individuals for depression, a decision was made to recruit an unselected sample of cancer survivors who expressed an interest in participating in the study.

Mailers containing a brief information flyer were sent to a total of 174 survivors of women’s cancer in the registry, followed by an email inquiry about their potential interest. Of these, 28 women responded, and all were deemed eligible and enrolled in the study. Interested individuals were asked to review and sign the consent form on a secure Qualtrics Web page. Research staff described the aims of the study and reviewed the study timeline with participants before they signed the consent. Participants then completed a Web battery of questionnaires and scheduled a coaching call (designed to last 30 min) to take place sometime within the next week, which marked the initiation of the intervention. After 6 weeks (postintervention), participants completed another battery of self-report measures online. Finally, participants completed the same battery of self-report measures 4 weeks after the intervention period. They also provided feedback about their experiences of using the app and coaching. Participants were compensated with a US $50 gift card for providing feedback. The data that support the findings of this study are available on reasonable request from the corresponding author.

### iCanThrive App

**Figure 1:**
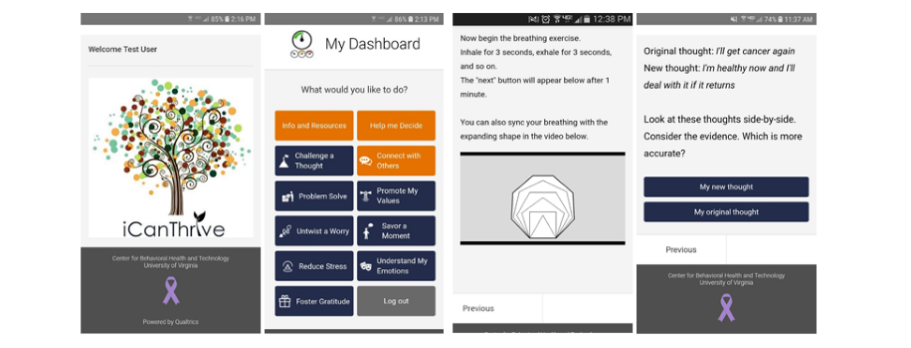
Screenshots of the iCanThrive app.

The iCanThrive app was available for public download on the Google Play Store in the United States. The app is user initiated, meaning that users launch and use the app when and where they desire. On launching the iCanThrive app, users are presented with a brief splash screen that contains the app logo and a lavender ribbon that is commonly used to raise awareness of cancer survivors (Figure 1, top left), before automatically directing them to the *My Dashboard* screen (Figure 1, top right). The depiction of a flourishing tree was chosen as the app logo to signify growth and skills acquisition as a means for empowering survivors of women’s cancer [70].

The core functionality of the app is composed of 8 exercise modules. Each module focuses on a specific aspect of mental health and well-being drawn from basic tenets of cognitive behavioral therapy (eg, reducing worry and problem solving), acceptance-based therapies (eg, mindfulness and emotional awareness), and positive psychology (eg, fostering gratitude and savoring positive experiences). See Table 1 for a description of the 8 iCanThrive modules and their objectives. Selecting a module immediately initiates an interactive exercise that guides the user through each step. For example, if a user selects the *Challenge a Thought* module from the *My Dashboard* screen, they are guided through an interactive exercise that leads them through the steps of writing down a negative or distorted recurring thought, determining what class of negative thought it falls under, and ultimately generating a new, alternative thought (Figure 1, bottom right). If a user selects the *Reduce Stress* module, they can engage in a diaphragmatic breathing exercise that presents them with a 1-min triangle breathing video they can use to synchronize their breath (Figure 1, bottom left). Users also complete a distress thermometer to assess change in psychosocial distress before and after completing the exercise. At the end of each module, users are given an option to log out of the app or navigate back to the *My Dashboard* screen. Exercises were designed to be completed within 2 min and require few instructions to complete.

**Table 1:** Description of iCanThrive modules and their objectives.

Module	Objective
Challenge a Thought	Enhances the ability to identify and challenge distorted thinking patterns. Guides users through a cognitive restructuring exercise.
Problem Solve	Promotes problem-solving skills. Users are led through an exercise that identifies ways to reach a goal while weighing the pros and cons of each strategy.
Untwist a Worry	Provides an interactive exercise to decrease worry. Users are led to consider the actual probabilities and costs of negative events happening and strategies to cope.
Reduce Stress	Increases relaxation skills. Users can choose to listen to short guided mindfulness audios or engage in an interactive diaphragmatic breathing exercise.
Foster Gratitude	Promotes a grateful outlook. Users are prompted to identify things they are grateful for and how to acknowledge them in daily life.
Promote My Values	Promotes awareness of values and ways to strive for fulfilling values in daily life.
Savor a Moment	Increases positive affect by leading user to recall and recount past positive experiences.
Understand My Emotions	Enhances emotional awareness. Users are led through an exercise to identify the emotions they are experiencing, the causal factors involved, and how their emotions are linked to their thoughts and behaviors.

The app contains additional functions that (1) allow users to learn more about the psychological constructs the app targets and connect users to trusted third-party sites (eg, American Cancer Society main website) that contain information about cancer support services and other electronic sources, (2) allow users to connect with other iCanThrive users through an anonymous discussion board, and (3) recommend 1 of the 8 exercise modules based on a series of questions that assess the user’s emotional and psychological state. Users were not specifically required to access these functions during the intervention period. As these functions were peripheral to the 8 exercise modules, their utilization is not reported. Each instance of an app launch was automatically logged and stored in the system supporting the app. This enabled us to track the total number of app launches across the study period.

### Phone Coaching

A manualized and detailed coaching protocol was developed based on the Efficiency Model of Support [63]. Similar coaching protocols have been implemented in other studies evaluating mental health apps [39]. The goals of coaching, which were listed in the coaching manual, are to address usability issues, increase engagement with the app, promote fit by assessing cancer survivors’ needs, promote knowledge of the skills found in the app, and encourage implementation of the skills in daily life [63]. Usability concerns include issues related to the usability of the intervention, fit of the intervention tool to one’s needs, knowledge of how to use the intervention, and implementation failures. The coaching manual provided a structure to coaching calls, including specific language to use and questions to ask. The structure that was prescribed by the coaching manual enabled coaches to methodically discuss each of the coaching goals. Coaches were instructed to focus on app-related issues and to refrain from doing more traditional counseling with participants. Participants were explicitly told that their coach is not trained in counseling or crisis management. An initial 30-min coaching call focused on orienting participants to downloading and using the app, setting expectations of the coach’s role, assessing how the app may meet participants’ needs, and building rapport. Participants were told that they could contact coaches at any time with any app-related questions via email or phone. Following the initial coaching call, participants received a text message (via Qualtrics Short Message Service tool) every week to remind them to try 2 new exercise modules in the app. Overall, 2 coaches with a bachelor’s degree were trained and monitored by the lead author (PC). Coaches received a detailed coaching manual and attended weekly supervision meetings throughout the duration of the trial. Finally, an unstructured 5-min phone call 4 weeks after the initial coaching call served as a check-in to make sure that participants did not have any lingering concerns or questions.

### Self-Report Measures

#### Psychosocial Outcomes

##### Depression

The Center for Epidemiologic Studies Depression Scale (CES-D [71]), 10-item version [72], was used to assess for symptoms of depression. The CES-D-10 is a well-validated and accepted measure of symptoms of depression in cancer populations [73,74]. Participants respond (0=rarely or none of the time and 3=all of the time) to 10 items that assess symptoms related to depression (eg, *I felt lonely* and *I felt depressed*). Participants are asked to base their responses on how they have felt over the past week. A cutoff score of 10 or greater, from a possible score range of 0 to 30, has been used to indicate a clinically significant level of depression in older adults [72].

##### Emotional Self-Efficacy

The Patient-Reported Outcomes Measurement Information System (PROMIS [75]) Self-Efficacy for Managing Emotions subscale (version 1.0, Short Form 4a) was used. PROMIS scales are well validated and widely used in health research. Participants respond (1=I am not at all confident and 5=I am very confident) to 4 items that assess behaviors related to emotion regulation and self-management (eg, *I can handle negative feelings* and *I can find ways to manage stress*). Scores range from 1 to 20. Consistent with PROMIS scoring recommendations, raw summed scores were converted into *t* scores for analyses, with higher scores indicating greater emotional self-efficacy.

##### Sleep Disturbance

The PROMIS [75] Sleep Disturbance subscale (version 1.0, Short Form 4a) was used to assess sleep. Participants respond (1=not at all and 5=very) to 4 items that assess sleep quality and related behaviors (eg, *My sleep was refreshing* and *I had difficulty falling asleep*). Scores range from 1 to 20. Higher scores indicate greater sleep disturbance. Raw summed scores were converted into *t* scores for analyses.

#### User Experience

The USE [76] short form was used to examine the usability and satisfaction of the iCanThrive app. It is composed of 21 items that assess user experience (eg, “I would recommend it to a friend,” “It is easy to learn to use it,” and “It is simply to use”), which comprise the domains of Usefulness, Ease of Use, Ease of Learning, and Satisfaction. Items are scored on a 7-point Likert scale (1=strongly disagree and 7=strongly agree). The USE measure is a well-validated scale that is commonly used to evaluate the user experience of mHealth interventions [77,78]. Moreover, 10 additional items were used to assess aesthetic appeal of the app, concerns about data privacy, usefulness of coaching calls, the degree to which iCanThrive meets a need for survivors of women’s cancer, and whether users would, in theory, be interested in being a coach for other survivors of women’s cancer. Participants rated each item on a 5-point Likert scale (1=not at all and 5=very). Table 2 contains the 10 additional items and their descriptive statistics.

**Table 2:** Feedback items (scale ranged from 1 to 5) and descriptive statistics (n=19).

Item	Values
1. How satisfied are you with the iCanThrive program in general? mean (SD)	4.06 (1.0)
2. How much did you like the way the iCanThrive program looked? mean (SD)	4.33 (0.69)
3. How much did the program keep your interest and attention? mean (SD)	3.56 (1.25)
4. How good of a fit was the program for you? mean (SD)	3.50 (1.34)
5. How worried were you about your privacy in using iCanThrive? mean (SD)	1.33 (0.69)
6. How likely would you be to continue using the program on your own? mean (SD)	3.00 (1.37)
7. How useful were the coaching phone calls in using the app? mean (SD)	4.22 (0.94)
8. How useful were the text message reminders in using the app? mean (SD)	4.13 (1.31)
9. How much do you think the iCanThrive program meets a need for women cancer survivors? mean (SD)	4.06 (1.21)
10. Would you be interested in being an iCanThrive coach for other women cancer survivors? (yes/no; N=19), n (%)	Yes=9 (47); no=10 (53)

### Data Analysis

Outcome data were stored in a secured Qualtrics server for highly sensitive data. Analyses were performed in SPSS version 25.

Study adherence and app usage were analyzed using descriptive statistics. A per-protocol analysis approach was adopted using paired *t* tests to analyze whether the use of iCanThrive was associated with changes in psychosocial outcomes (ie, depression, emotional self-efficacy, and sleep disturbance) before versus after the intervention period. Paired *t* tests were also used to examine whether there was a significant difference in psychosocial outcomes from baseline to the 4-week follow-up. User experience data were analyzed descriptively by obtaining means and standard deviations.

## Results

### Study Adherence and App Usage

Of 28 cancer survivors, 20 (71%) completed the postintervention assessment, and 19 cancer survivors completed the 4-week follow-up assessment. Of the 8 individuals who dropped out of the study, 5 did not engage in the initial coaching call and, therefore, did not initiate treatment, and 3 individuals lost contact after completing the coaching call. Thus, of 23 cancer survivors who initiated treatment, 20 (87%) completed the postintervention assessment, and 19 (83%) completed the 4-week follow-up assessment. The iCanThrive app was launched a median of 20.5 times (mean 23.2, SD 16.8; range 1-59; IQR=25) over the 6-week intervention period, which is generally higher than other apps that have produced significant improvements in mental health outcomes [79,80]. The median duration of use per app launch was 2.1 min. A total of 3 participants requested additional contact with coaches during the course of the study. Of those, 2 participants wanted to clarify the timing of when they should try new exercise modules, and 1 participant asked if they could tell a friend about the app.

### Psychosocial Outcomes

Table 3 contains descriptive statistics of psychosocial outcomes. There was a significant reduction in symptoms of depression from baseline to postintervention (t19=2.22; *P*=.04; 95% CI 0.08 to 2.72). Despite survivors reporting a lower level of depression symptoms at the 4-week follow-up than at baseline, this effect did not reach significance (t16=0.82; *P*=.42; 95% CI −0.93 to 2.11). There was no significant difference in symptoms of depression from postintervention to the 4-week follow-up (t18=1.13; *P*=.27; 95% CI −0.63 to 2.11). Among those who completed the 6-week intervention (n=20), at baseline, a total of 6 individuals had depression scores at or above the CES-D-10 cutoff (≥10) for clinically significant depression. At postintervention, 1 individual was above this threshold. At the 4-week postintervention follow-up, a total of 3 individuals were above this threshold.

**Table 3:** Means and standard deviations of psychosocial outcomes at each time point.

Outcome	Baseline (n=28), mean (SD)	Postintervention (n=20), mean (SD)	4-week follow-up (n=19), mean (SD)
Depression symptoms	6.25 (3.85)	4.85 (2.92)	5.47 (3.78)
Emotional self-efficacy	48.37 (6.50)	50.23 (5.44)	50.59 (6.29)
Sleep disruption	48.47 (6.88)	44.37 (8.16)	43.92 (6.74)

There was a slight increase in emotional self-efficacy from baseline to postintervention, although this effect did not reach significance (*t*_19_=1.33; *P=*.20; 95% CI −4.79 to 1.07). Despite women reporting greater emotional self-efficacy at the 4-week follow-up than at baseline, this effect did not reach significance (*t*_18_=1.56; *P=*.14; 95% CI −5.35 to 0.79). Finally, there was a significant reduction in sleep disruption from baseline to postintervention (*t*_19_=3.41; *P=*.003; 95% CI 1.59 to 6.62), and there continued to be a significant difference in sleep disruption from baseline to the 4-week follow-up (*t*_18_=3.71; *P=.*002; 95% CI 1.97 to 7.11).

### User Experience

Overall, survivors of women’s cancer reported very high levels of ease of use (mean 6.12 out of 7, SD 0.91) and ease of learning (mean 6.49 out of 7, SD 0.71) the iCanThrive app. They also reported an acceptable level of usefulness (mean 4.87 out of 7, SD 1.55) and a high level of satisfaction (mean 5.19 out of 7, SD 1.36) of the app. As seen in Table 2, they also reported that they generally liked how the app looked (mean 4.33 out of 5, SD 0.69), that the app was at least somewhat effective at keeping their attention (mean 3.56 out of 5, SD 1.25), and that the app strongly endorsed the utility of phone coaching to supplement their use of the app (mean 4.22 out of 5, SD 0.94). Mean scores for the overall fit of the intervention (mean 3.50 out of 5, SD 1.34) and interest in continuing to use the app (mean 3.00 out of 5, SD 1.37) indicated generally favorable ratings, although the standard deviations suggest a large amount of variance in how users rated these items. Individuals also reported having little concern over privacy issues of the app (mean 1.53 out of 5, SD 0.52). Finally, users reported, on average, that iCanThrive meets an important need for survivors of women’s cancer (mean 4.06 out of 5, SD 1.21). Roughly half (9/19, 47%) of the women who completed the study indicated that they would be willing to serve as a coach for other survivors of women’s cancer to help them use the app.

## Discussion

### Principal Findings

Overall, the app usage metrics and patient-reported outcomes from this pilot study support the use of iCanThrive as a clinical intervention in survivors of women’s cancer. To our knowledge, there are no published trials of app-based interventions with phone coaching that specifically target mental health outcomes among survivors of women’s cancer. The findings of this study suggest that survivors of women’s cancer, many of whom live in nonmetropolitan areas, can engage with and benefit from a mobile intervention. Few trials of app-based interventions report follow-up data on outcomes. In this study, despite symptoms of depression being lower at the 4-week follow-up assessment than at baseline, the difference was not significant. It is likely that this effect would be significant with a larger sample size and with a clinically depressed sample. However, the sustainability of these gains should be a focus of development for future interventions. For example, although women used iCanThrive over 6 weeks, a longer intervention period could yield larger and long-lasting gains.

In general, feedback scores from women was largely positive, although future iterations of the app should consider some changes. In this study, feedback scores were lowest for items related to fit and long-term engagement after the trial period. Sustained engagement is a widespread issue facing mobile and app-based interventions [49], many of which rely on users to initiate. An alternative method of content delivery involves leveraging adaptive designs. For example, a Just-in-Time Adaptive Intervention [81] aims to provide the right type and amount of support, at precisely the right time, by continually assessing and adaptive to the user’s state. Future trials of iCanThrive may evaluate the utility of continuously assessing a user’s emotional state passively through mobile phone sensors [82] or via self-report instruments to trigger interventional content when the user is most vulnerable (eg, a user is asked to engage in a breathing exercise when their emotional state is on a downward trajectory). In addition, future work should explore integrating components to the intervention that assist the user in making decisions about what exercises to engage in. For example, some mental health apps have evaluated the usefulness of automated recommender systems that encourage users to try intervention content that most closely matches their current needs [83]. The goals of these types of strategies are to promote fit and engagement with digital interventions.

By providing a provision of light human support, iCanThrive is different from most other app-based mental health interventions, and the use of phone coaching likely enhanced users’ engagement with the app (based on app usage and relatively low dropout rates compared with other digital intervention studies). Although the decision to add phone coaching was based on the communication preferences in women versus men [65], not all women will use an app-based intervention the same way. Some may benefit from phone coaching, whereas others may not. Thus, although women in this trial strongly endorsed the utility of phone coaching, future trials should consider whether to provide phone coaching to a subset of women who need it most. For example, using a Sequential, Multiple Assignment, Randomized Trial [84], it may be possible to identify individuals who struggle to engage with the app and provide them with additional resources. Addressing support needs on an individual basis will maximize the scalability and impact of an app-based intervention by making efficient use of available resources (eg, money and coach’s time). Furthermore, future studies may wish to explore the benefits of training lay individuals to become coaches, as almost half of the participants in this trial reported that they would be interested in coaching future survivors of women’s cancer. Training individuals who have completed the intervention to become coaches could increase the scalability of a digital intervention while promoting engagement.

### Limitations

This study and its findings should be interpreted in light of several limitations. Given the size and characteristics of the current sample, these findings may not be generalizable to subsets of women with a specific form of cancer or phase. The participants in the study were not selected based on a threshold of depressive symptoms, and future studies may wish to recruit a clinically depressed sample. One might expect the impact of iCanThrive on symptoms of depression, emotional self-efficacy, and sleep disturbance to be even greater among those with a clinical level of depression. Findings related to the potential impact of iCanThrive on symptoms of depression and sleep disruption should also be replicated in a larger sample of survivors of women’s cancer. For example, in this study, it was found that depression symptoms, although lower at the 4-week postintervention follow-up (vs baseline), was not significantly different than at baseline, which may be attributed to the small sample size and limited power. We urge caution in using pilot studies to guide power calculations for larger trials [85]. Furthermore, it is important to consider other possible outcomes to assess, such as social functioning and positive affect. It will also be important to evaluate iCanThrive in a randomized controlled trial, as this study used a single-group, pre-post design. Thus, it is important to not overinterpret the findings of this study because of the absence of a control condition. Finally, it will be important for future studies to collect in-depth qualitative feedback to improve the iCanThrive app and phone coaching protocol.

### Conclusions

Taken together, these findings support the acceptability and preliminary efficacy of iCanThrive for reducing mood symptoms in survivors of women’s cancer. There were significant reductions in symptoms of depression and sleep disruption from baseline to postintervention, which supports the potential usefulness of examining iCanThrive in future trials. In addition, participants found the app-based intervention to be easy to use and generally useful for improving their mood, which was consistent with data on user engagement. They also reported high levels of satisfaction with iCanThrive and felt that it met an important need for survivors of women’s cancer.

## Abbreviations

CES-D: Center for Epidemiologic Studies Depression Scale
mHealth: mobile health
PROMIS: Patient-Reported Outcomes Measurement Information System
RUCA: rural-urban commuting area
